# Spiral laser scanning photoacoustic microscopy for functional brain imaging in rats

**DOI:** 10.1117/1.NPh.11.1.015007

**Published:** 2024-02-09

**Authors:** Mohsin Zafar, Laura Stone McGuire, Seyed Mohsen Ranjbaran, James I. Matchynski, Rayyan Manwar, Alana C. Conti, Shane A. Perrine, Kamran Avanaki

**Affiliations:** a University of Illinois at Chicago, Department of Biomedical Engineering, The Richard and Loan Hill, Chicago, Illinois, United States; b University of Illinois at Chicago, Department of Neurosurgery, Chicago, Illinois, United States; c John D. Dingell Veterans Affairs Medical Center, Detroit, Michigan, United States; d Wayne State University School of Medicine, Department of Neurosurgery, Detroit, Michigan, United States; e Wayne State University School of Medicine, Department of Psychiatry and Behavioral Neurosciences, Detroit, Michigan, United States; f University of Illinois at Chicago, Department of Dermatology, Chicago, Illinois, United States

**Keywords:** photoacoustic microscopy, optical resolution photoacoustic microscopy, laser scanning, spiral scanning, rat brain, functional imaging, cerebral hemodynamics, whisker stimulation, electrical stimulation, brain injury

## Abstract

**Significance:**

There are many neuroscience questions that can be answered by a high-resolution functional brain imaging system. Such a system would require the capability to visualize vasculature and measure neural activity by imaging the entire brain continually and in rapid succession in order to capture hemodynamic changes. Utilizing optical excitation and acoustic detection, photoacoustic technology enables label-free quantification of changes in endogenous chromophores, such as oxyhemoglobin, deoxyhemoglobin, and total hemoglobin.

**Aim:**

Our aim was to develop a sufficiently high-resolution, fast frame-rate, and wide field-of-view (FOV) photoacoustic microscopy (PAM) system for the purpose of imaging vasculature and hemodynamics in a rat brain.

**Approach:**

Although the most PA microscopy systems use raster scanning (or less commonly Lissajous scanning), we have developed a simple-to-implement laser scanning optical resolution PAM system with spiral scanning (which we have named “spiral laser scanning photoacoustic microscopy” or sLS-PAM) to acquire an 18 mm diameter image at fast frame rate (more than 1 fps). Such a system is designed to permit continuous rat brain imaging without the introduction of photobleaching artifacts.

**Conclusion:**

We demonstrated the functional imaging capability of the sLS-PAM system by imaging cerebral hemodynamics in response to whisker and electrical stimulation and used it for vascular imaging of a modeled brain injury. We believe that we have demonstrated the development of a simple-to-implement PAM system, which could become an affordable functional neuroimaging tool for researchers.

## 1 Introduction

Photoacoustic imaging (PAI) is an emerging hybrid imaging modality, combining the advantages of optical contrast with acoustic penetration.^1^^–^^3^ PAI has been used extensively in preclinical,^4^^–^^10^ and more recently, clinical studies.^11^^–^^21^ In PAI, upon nanosecond laser irradiation of tissue, chromophores, such as oxy-hemoglobin (${\mathrm{HbO}}_{2}$) and deoxy-hemoglobin (Hb), absorb energy and generate photoacoustic waves through the thermoelastic effect.^1^^,^^22^^–^^25^ The generated waves are detected by ultrasound transducers and processed by an image reconstruction algorithm. PAI has previously been used to provide high-resolution structural and functional images of brain vasculature in small and large animal models.^26^^–^^28^ Many neuroscience questions can be answered by a high-resolution functional brain imaging system.^22^^,^^29^^,^^30^ For functional imaging, PAI relies on the same principle as that of functional magnetic resonance imaging, where neural activity is indirectly captured through imaging of cerebral hemodynamic fluctuations (i.e., changes in oxygen demand). Many applications of PAI for functional brain imaging have been reported. For example, Wang et al.^4^ imaged cerebral hemodynamic changes in the rat brain in response to whisker stimulation, hyperoxia and hypoxia; Nasiriavanaki et al.^22^^,^^31^ developed a photoacoustic computed tomography (PACT) system to monitor the cortical resting state functional connectivity in the mouse brain;^1^ Kang et al. imaged neuronal activity during seizures in the mouse brain;^32^ Liao et al.^33^ imaged hemodynamic changes in the rat brain in response to forepaw electrical stimulation; and Janggun et al.^34^ developed a functional PAI system to monitor regional activation in the rat brain induced by cocaine.

There are two major implementations of PAI: PACT and photoacoustic microscopy (PAM), the latter of which is further divided into two categories, acoustic resolution-PAM and optical resolution PAM (OR-PAM), based on how the focus is achieved.^1^^,^^35^ Although PACT is used for imaging hemodynamic parameters in larger vessels in deeper regions,^4^^,^^22^^,^^36^^–^^39^ PAM is used for imaging fine vessels, i.e., capillaries in shallow regions.^40^^–^^45^ Analysis of hemodynamic changes in capillaries, as compared to larger vessels in the brain, provides a more detailed understanding of brain functionality.

Early versions of OR-PAM used 2D galvo scanners to provide an imaging area of ∼6  mm diameter in about 2 min.^46^ By incorporating faster scanning hardware, such as microelectromechanical system mirrors, second generation OR-PAM systems became faster. For example, Yao et al.^47^ developed an OR-PAM system able to image an area of $2.5\times 4\;{\mathrm{mm}}^{2}$ in 37 s; Lan et al.^48^ developed a hexagon-based OR-PAM system that imaged an area of $12\times 15\;{\mathrm{mm}}^{2}$ in 12 s; Li et al.^49^ developed an OR-PAM system using a combination of mechanical and optical scanning able to image an area of $10\times 8\;{\mathrm{mm}}^{2}$ within 180 s; Lee et al.^50^ introduced a fully waterproof 2D galvanometer scanner able to image $8\times 13\;{\mathrm{mm}}^{2}$ in 100 s; Zhong et al.^51^ utilized a cylindrically focused transducer imaging 76  μm×4.5  mm slices simultaneously to capture an area of $4.5\times 1\;{\mathrm{mm}}^{2}$ in 1 s. Chen et al.^52^ developed polygonal scanning method to image an area of $12\times 12\;{\mathrm{mm}}^{2}$ in 5 s.

In the implementation of OR-PAM, sometimes an unfocused ultrasound transducer is kept stationary, at an inclined angle, and a laser light is raster scanned point-by-point by means of a 2D galvanometer within the field-of-view (FOV) of the transducer. While not a new type of OR-PAM, this method has been referred to as laser scanning PAM (LS-PAM) in the literature.^10^^,^^52^ LS-PAM has lower signal-to-noise ratio (SNR) compared to other configurations of OR-PAM, mainly due to three reasons: (i) noncoaxial opto-acoustic configuration, (ii) unfocused transducer for detection, and (iii) transducer placement further away from the imaging target.^53^

The three main scanning configurations that have been explored for optical imaging are raster scanning, Lissajous scanning, and spiral scanning.^54^ In raster scanning, the imaging beam or stage moves in a back-and-forth pattern across the sample in a grid-like fashion. Although raster scanning allows for precise control over the scanning path and image acquisition, it is slower compared to other scanning techniques and may cause motion artifacts because of the need for stopping and starting at the end of each line. This stop-start movement (fly-back) can result in misalignment between consecutive scan lines and can lead to image distortion or artifacts. Some implementations of raster scanning, such as bidirectional triangular and sinusoidal, have improved on the speed of image acquisition.^55^^,^^56^ However, triangular scans still suffers from interruption times (phase lag) and sinusoidal scans display deformations of the image at the margins because of nonlinearities.^55^ A thorough comparison of raster scanning functions (triangular, sinusoidal, and sawtooth) is explored in a series of papers.^54^^–^^56^ Lissajous scanning involves using orthogonal oscillations to scan the imaging beam across the sample in a two-dimensional pattern using sinusoidal oscillations in both the x and y axes to create a complex scanning trajectory resembling Lissajous figures. It is faster than raster scanning, as it avoids the need for repetitive movements, and avoids motion artifacts, but requires additional complexity in the scanning mechanism and control system. Its scanning pattern requires visiting the same regions on multiple passes, which can increase photobleaching, and requires additional postprocessing because of the nonlinearity of pixel spacing.^57^ Spiral scanning involves a continuous spiral motion of the imaging beam or stage from the center towards the outer edges of the sample.^58^ The entire FOV is covered in a single continuous motion without the need for repetitive movements. It enables faster image acquisition compared to raster scanning, as it eliminates back-and-forth movements, minimizes exposure time at each point, and reduces motion artifacts. Spiral scanning also provides a more even distribution of sampling points across the entire scanned area and the density of points remains relatively consistent throughout the spiral trajectory. More details on different implementations of spiral scanning including mathematical details and potential areas for distortion are discussed in Refs. 58 and 59. We previously studied brain activities by imaging the main functional cortical regions using ring array PACT;^22^^,^^60^ one of the motivations for the current study was to enable imaging of cortical subregions within the main functional regions. This has been accomplished by developing a robust, sufficiently large FOV, fast system with reasonable resolution.

## 2 Material and Methods

Although the most PA microscopy systems use raster scanning (or less commonly Lissajous scanning), here we develop a simple-to-implement laser scanning OR-PAM system with spiral scanning (sLS-PAM) to acquire a large image at a fast frame rate. The design requirements include ability to continuously image the same region on the brain without the introduction of photobleaching artifacts and a high sensitivity acoustic detection unit.

### 2.1 Principle of Spiral Scanning

**Fig. 1:**
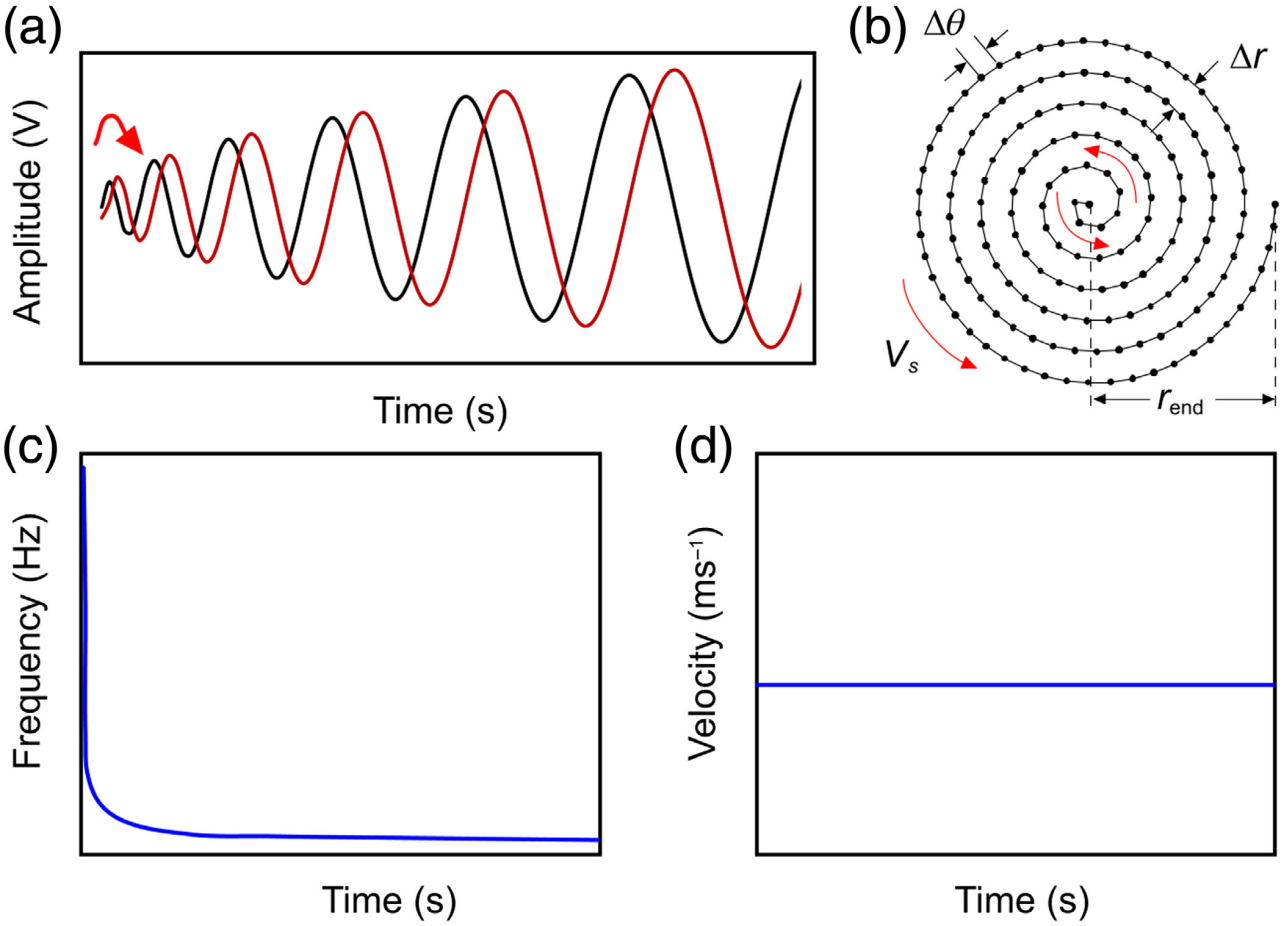
Description of spiral scanning implementation: (a) sinusoidal voltage waveforms for driving x (black) and y (red) mirrors of the galvanometer; (b) spiral scan pattern generated by sinusoidal voltage waveforms; (c) frequency of sinusoidal voltage as a function of time; and (d) velocity along the spiral as a function of time. $V_{s}$, spiral scan velocity; Δθ, spiral pitch in angular dimension θ (along the spiral); Δr, sampling pitch in radial dimension r; and $r_{\text{end}}$, radius of spiral scan circular field of view. Red arrows denote start of the scan or direction of travel. All scan patterns are undersampled for visual clarity.

Spiral scanning is a natural solution to mitigate the scanning inefficiencies of raster scanning methods, due to its ability to form a continuous scan pattern. Spiral scanning can be implemented with different spiral scan functions,^58^ including constant linear velocity $V_{s}$. To construct a system with constant linear velocity, a spiral scanning pattern was constructed by driving x and y mirrors of the galvanometer with x(t)=r(t)cos(ν(t)) and y(t)=r(t)sin(ν(t)) as shown in red and black in Fig. 1(a)(i), respectively. Here ν(t) is the frequency at time t, as shown in Eq. (1); r(t) is amplitude at time t, shown in Eq. (2), where Δr is the spiral (sampling) pitch normal to the radial dimension:

$$\nu (t)=\sqrt{\frac{V_{s}\times 4\pi t}{\Delta r}},$$ (1)

$$r(t)=\sqrt{\frac{V_{s}\Delta rt}{\pi }},$$ (2)

$$V_{s}=\frac{d\nu (t)}{dt}r(t)=\omega (t)r(t).$$ (3)

**Fig. 2:**
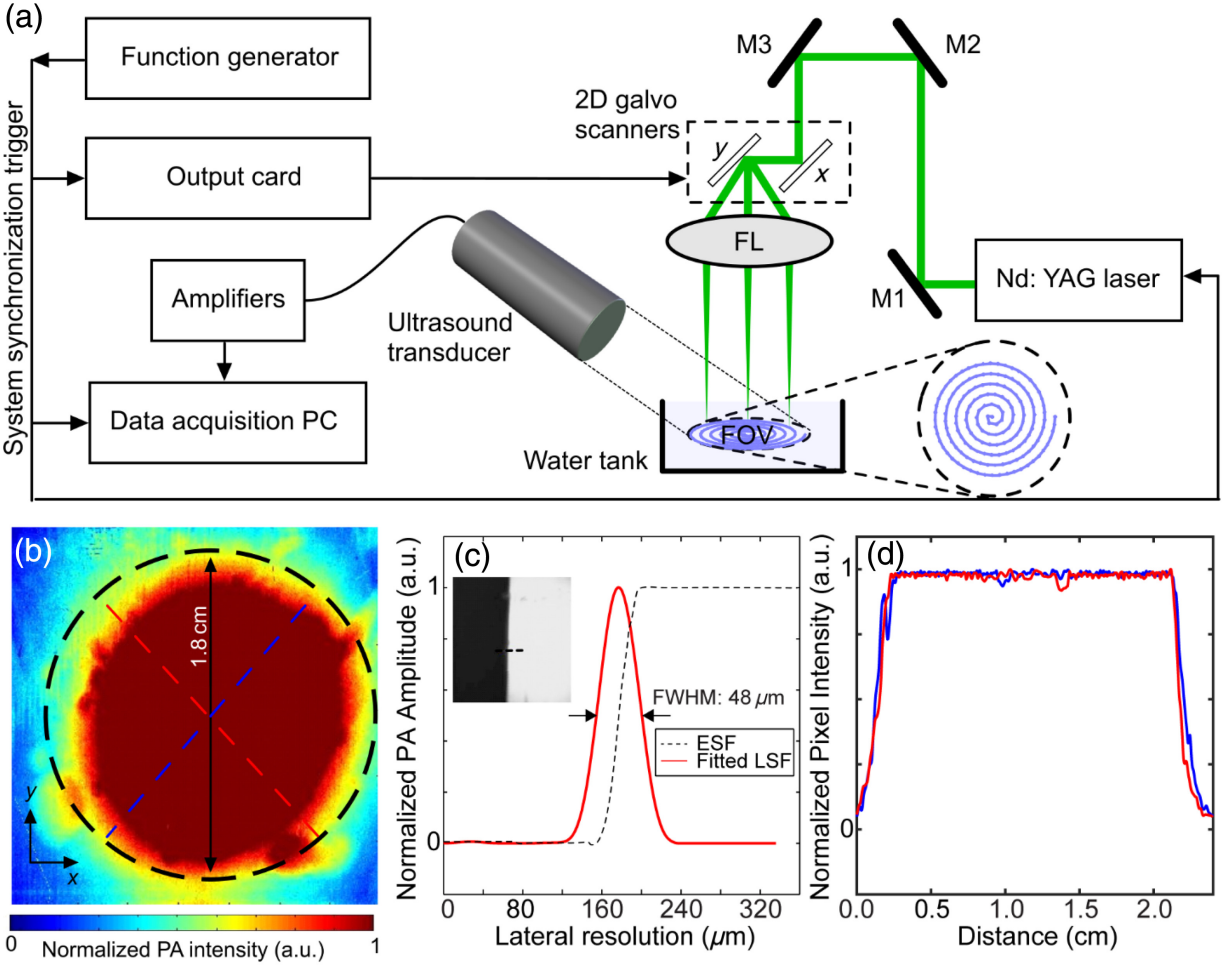
Experimental setup and specifications of wide-field sLS-PAM system: (a) schematic of the system, (b) detection sensitivity map obtained by imaging a piece of black tape, (c) lateral resolution obtained by imaging a sharp blade edge, and (d) two orthogonal intensity profiles across the dotted lines shown in (b). M1–M3: silver mirrors; FL; focusing lens; PC, personal computer; FOV, field of view; FWHM, full-width half-maximum; LSF, line spread function; ESF, edge spread function.

We use the term linear velocity $V_{s}$ (in units of m/s) to mean the velocity along the path of the scanner and differentiate it from angular velocity ω(t) (in units of rad/s). More details on the use and derivation of Eqs. (1)–(3) can be found in Ref. 59. Equation (3) expresses the relationship between $V_{s}$ and ω(t) of the spiral pattern. $V_{s}$ is held constant to maintain homogenous spiral pitch or points separation [Fig. 2(d)]; therefore, the frequency of scanning decreases rapidly as we scan outwards [Fig. 2(c)]. Implementing this decrease in frequency is straightforward using galvanometers, because they can scan very fast, as needed for the beginning of the spiral scan, but can be precisely programmed to slow as the scanning extends outwards. Δθ and Δr are the spiral pitch along the angular and radial axes of the spiral scan, respectively, and are expressed as

$$\Delta \theta =\frac{V_{s}}{f_{A\text{-}\text{scan}}},$$ (4)

$$\Delta r=\frac{\pi r^{2}(t)}{tV_{s}}.$$ (5)

Equation (4) is analogous to sampling pitch along the fast axis of a raster scan^59^ where $f_{A\text{-}\text{scan}}$ is the frequency of generating one A-scan. Equation (5) was derived by solving Eq. (2) for Δr. To achieve a uniform isotropic transverse sampling, Δθ was set equal to Δr. A more thorough explanation of spiral scanning can be found in Refs. 58 and 59.

### 2.2 System Development and Characterization

Figure 2(a) shows the schematic of the sLS-PAM system. A pulsed Nd:YAG laser (VPGL-G-20, V-gen, Tel Aviv, Israel) was used as an optical excitation source with a wavelength of 532 nm and a pulse repetition rate of up to 700 kHz. Through a combination of three 90 deg reflective mirrors (MPD019-G01, Thorlabs, Newton, New Jersey, United States), the light from the laser was focused onto a 2D-galvanometer (GVS 202-2D, Thorlabs, Newton, New Jersey, United States). The specifications for the galvanometer are given in Table 1. The XY mirrors in the galvanometer were controlled and operated by a LabVIEW program interfaced with a terminal block (BNC 2110, National Instruments), to generate two orthogonal sinusoidal signals [see Fig. 1(a)] to create a spiral scanning pattern [see Fig. 1(b)].

**Table 1:** Specifications of galvo scanner.

Parameter	Value
Optical scan angle range	−25 deg to + 25 deg
Mechanical scan angle range	−12.5 deg to +12.5 deg
Scan frequency	≥1 kHz for mechanical scan angle < 0.2 deg
	≤250 Hz for mechanical scan angle > 0.2 deg
Wavelength	400 to 700 nm
Input voltage range	± 6.25 V
Input beam diameter	1 to 5 mm

An output card was used to generate a frame trigger signal to initiate the start and end of data acquisition. The laser irradiation, spiral scanning, and frame trigger were synchronized by a square shaped signal produced by an external function generator (ATF20B, ATTEN instruments, United States). After the galvanometer, the spirally scanned laser beam was focused onto the imaging target through an achromatic doublet lens (AC127-075-A, Thorlabs, Newton, New Jersey, United States) with a focal length of 7.5 cm following its use in Refs. 61–63. The AC127-075-A doublet lens was carefully chosen to meet the requirements for continuous imaging (minutes) without damage to the live target or the lens. This lens has a high damage threshold which guarantees its endurance during long (few seconds to few minutes) functional imaging sessions. The relatively low NA lens we have used guards against photobleaching while still providing acceptable resolution (<48  μm). Although imaging with a higher NA lens can provide detail on the finest capillaries, in our experience, upstream capillaries (proximate to veins and arteries) provide more consistent results for measuring hemodynamic change, possibly because hemoglobin volume in the smallest capillaries are not as large as in the upstream capillaries. A water tank was constructed with a Saran wrapped bottom side. The imaging target was pushed up against the Saran wrap to avoid any air gaps. To ensure a perfect coupling, ultrasound gel was used between the Saran wrap and the imaging target.

Upon laser irradiation, the induced PA signals were detected by an ultrasound transducer (Centrascan C306, Olympus NDT; central frequency: 2.25 MHz; −6  dB bandwidth 66%). Centrascan and Videoscan transducers from Olympus are widely used for implementing PAM. The Centrascan transducers, with a piezocomposite element, provide excellent sensitivity with a high SNR. The backing layer in these transducers is composed of a highly attenuative, high-density material that controls the vibration of the transducer by absorbing the energy radiating from the back face of the active element. There is a mismatch in acoustic impedance between the element and the backing, leading to more sound energy reflected forward into the imaging target, which results in greater sensitivity. By comparison, the Videoscan transducer is heavily damped due to good acoustic impedance matching with backing material, which provides a broader bandwidth, but lower sensitivity. The largest available Centrascan transducer is 5 MHz. We ran tests on both the 2.25 and 5 MHz transducers to check differences in resolution and signal strength. The tests demonstrated that the 2.25 MHz model provides 20% higher sensitivity than the 5 MHz, with only 5% loss in resolution. This low-frequency transducer selection is also beneficial for PAM imaging through rodent skull.

The transducer was placed in the water tank, 40 mm away from the imaging target at an angle of 30 deg. Different angles of the transducer give different sensitivity distribution maps and affect the depth shift in the PA signal. An empirical study was performed where using a goniometer, the angle and distance of the transducer was iteratively varied until a maximum area of uniform sensitivity was achieved. Maximum area of uniform sensitivity was determined by measuring full-width half-maximum (FWHM) across two perpendicular line profiles of the imaging area following.^64^ A maximum uniform sensitivity area (∼9  mm radius) was achieved when the transducer was placed 30 deg from the y axis and 40 mm from the imaging object [see Figs. 2(a) and 2(b)]. Once the transducer position was fixed with these parameters, it was not changed for all the other studies presented in this manuscript.

A variable gain voltage amplifier with a maximum gain of up to 80 dB (Model 351A, Analog Modules Inc., Longwood, Florida, United States) was used to amplify the detected PA signals. The signals were then digitized at a sampling rate of 100  MS/s using a data acquisition system (GaGe CSE1642, Vitrek Corporation, Poway, California, United States). The data were streamed in real time. The angle within which the galvo mirrors moved, the FOV of the transducer, and the focal length of the objective lens determine a 18 mm diameter FOV, inside which the lateral resolution was within 6 dB fall-off. The FOV and sensitivity map were measured by imaging a piece of flat black tape [see Fig. 2(b)]. To characterize the lateral resolution of the system, we imaged a sharp blade (Rexbeti, Auburn, Washington, United States) [see Fig. 2(c)], where the resolution was measured as 48  μm by calculating the FWHM of the Gaussian profile across the green line shown in Fig. 2(c); the coarse lateral resolution is partially due to transducer frequency, which filters out PA signals generated from finer structures. Two orthogonal intensity profiles [dotted lines shown in Fig. 2(b)] are used to characterize the sensitivity map [Fig. 2(d)].

For all subsequent experiments, each frame consisted of 160,000 scanning points, scanned with sampling pitch (Δθ) and spiral pitch (Δr) of 40  μm (beam size on the surface of the imaging target was 16.9  μm). The sinusoidal signals applied to the galvanometer scanners had progressive voltage values and frequency [see Fig. 1(a)], which generated a constant velocity ($V_{s}$) of 8  m/s [see Fig. 1(d)]. The segment size per point acquired was 500 samples and the total data set acquired per frame was 200 MB [160,000×500×14  bits (2 bytes)]. For all the animal studies, pulse repetition rate of the laser was set to either 200 kHz (0.8 s acquisition/frame) or 400 kHz (0.4  s/frame) to scan one spiral frame with a diameter of ∼18  mm.

### 2.3 Depth Correction Algorithm

**Fig. 3:**
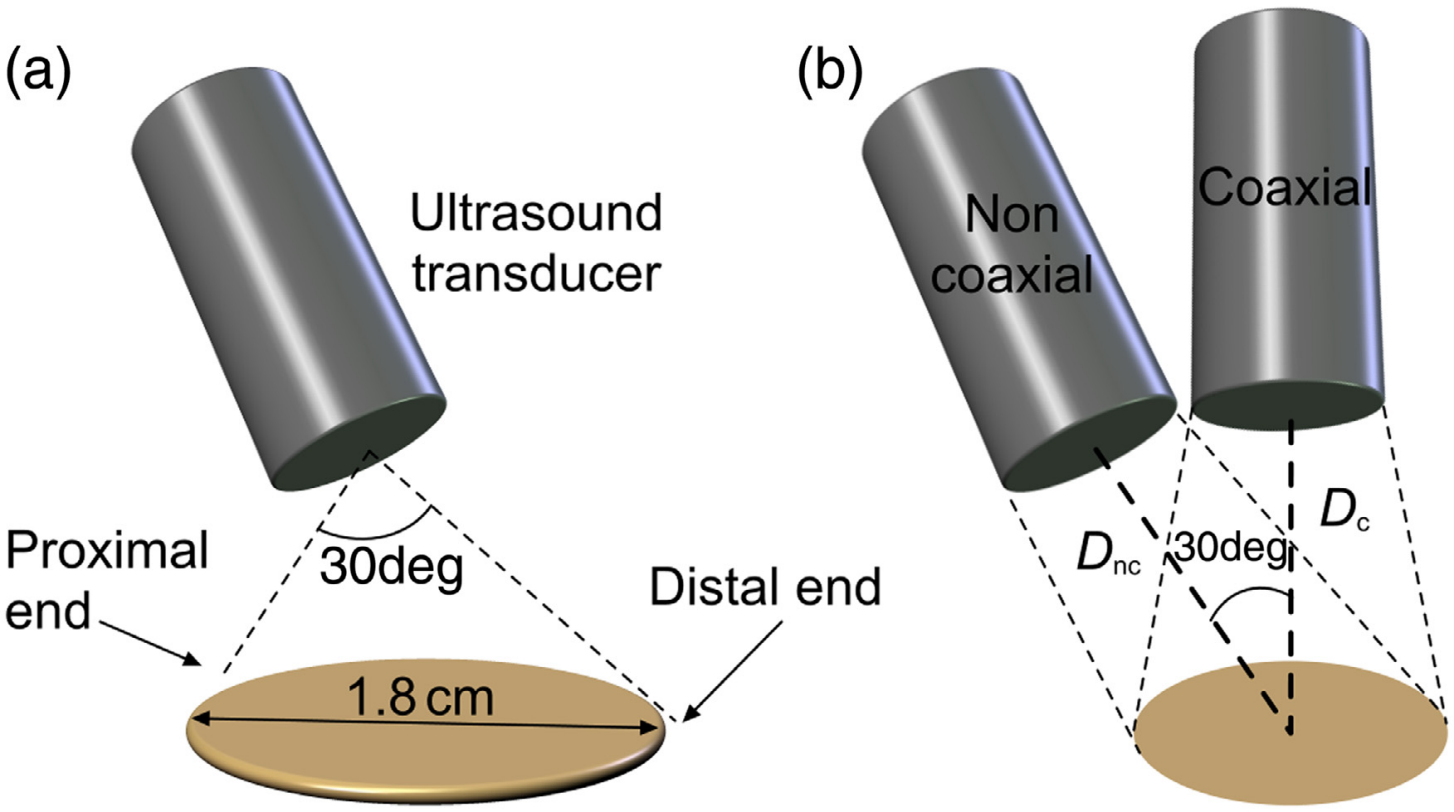
Schematic demonstrating depth misalignment and correction in sLS-PAM. (a) Angled position of the transducer leading to depth misalignment. (b) Virtually shifted angled transducer from noncoaxial to axial position. $D_{\mathrm{nc}}$, distance from the noncoaxial transducer to the center of the imaging area. $D_{c}$, corrected distance from theoretical coaxial position of the transducer to the center of the imaging area.

One limitation of an sLS-PAM setup is the noncoaxial arrangement of optical and acoustic focus because the transducer is placed at an angle [Fig. 3(a)]. Due to angled transducer placement, the data acquired are proportionally shifted in the axial direction, leading to some degree of depth misalignment. In our setup, the transducer is placed at an angle of 30 deg from the target, as shown in Fig. 3(a). Due to angled transducer placement, the proximal end of the imaging target is closer than the distal end. Therefore, the PA signals generated from the proximal end of the target reach the ultrasound transducer earlier than the PA signals from the distal end. This introduces a gradual time delay, because the points are scanned from proximal to distal end, in turn affecting axial/depth alignment of the images.

**Fig. 4:**
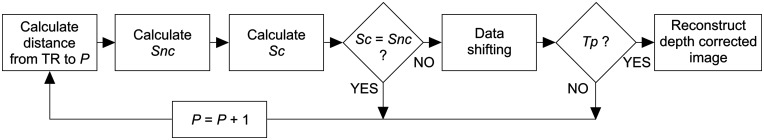
Flowchart for depth correction algorithm. TR, transducer; P, a single point in the spiral scan; Snc and Sc, sample number computed from the center of angled transducer to point P in spiral scan. $T_{p}$, total number of points in spiral scan.

We developed a depth correction algorithm to virtually shift the transducer from the angled (i.e., noncoaxial) position to a coaxial position [Fig. 3(b)]. In this algorithm, the distance ($D_{\mathrm{nc}}$) from each point (P) of the spiral scan pattern was calculated from the center of the angled transducer. Similarly, the distance ($D_{c}$) from the center of the coaxial position of the transducer was computed. The relationship between $D_{c}$ and $D_{\mathrm{nc}}$ is $D_{c}=D_{\mathrm{nc}}\;\cos\;\varphi$, where φ is the angle between the two positions. Based on $D_{\mathrm{nc}}$ and $D_{c}$, the sample numbers in which we expected to see PA signals were calculated, we call them noncoaxial (Snc) and coaxial samples (Sc), respectively. If Snc and Sc were not equal, data were shifted to match these sample numbers. (The order of the samples was shifted from the order in which they actually reached the target to the order in which they would have reached the target had they been coaxial.) Note that our depth correction algorithm only shifts the data without modifying it. The pixel values of the image before and after image correction were essentially the same (because the depth correction algorithm does not change pixel values). The data correction algorithm is summarized in a flowchart in Fig. 4. Subsequently, to evaluate the performance of the algorithm, a parameter called “depth anomaly,” DA was defined. To calculate DA, an axial line profile was acquired from the proximal to the distal end of the image. The slope of the line profile was then used to calculate DA. The steeper the slope is, the higher DA is.

**Fig. 5:**
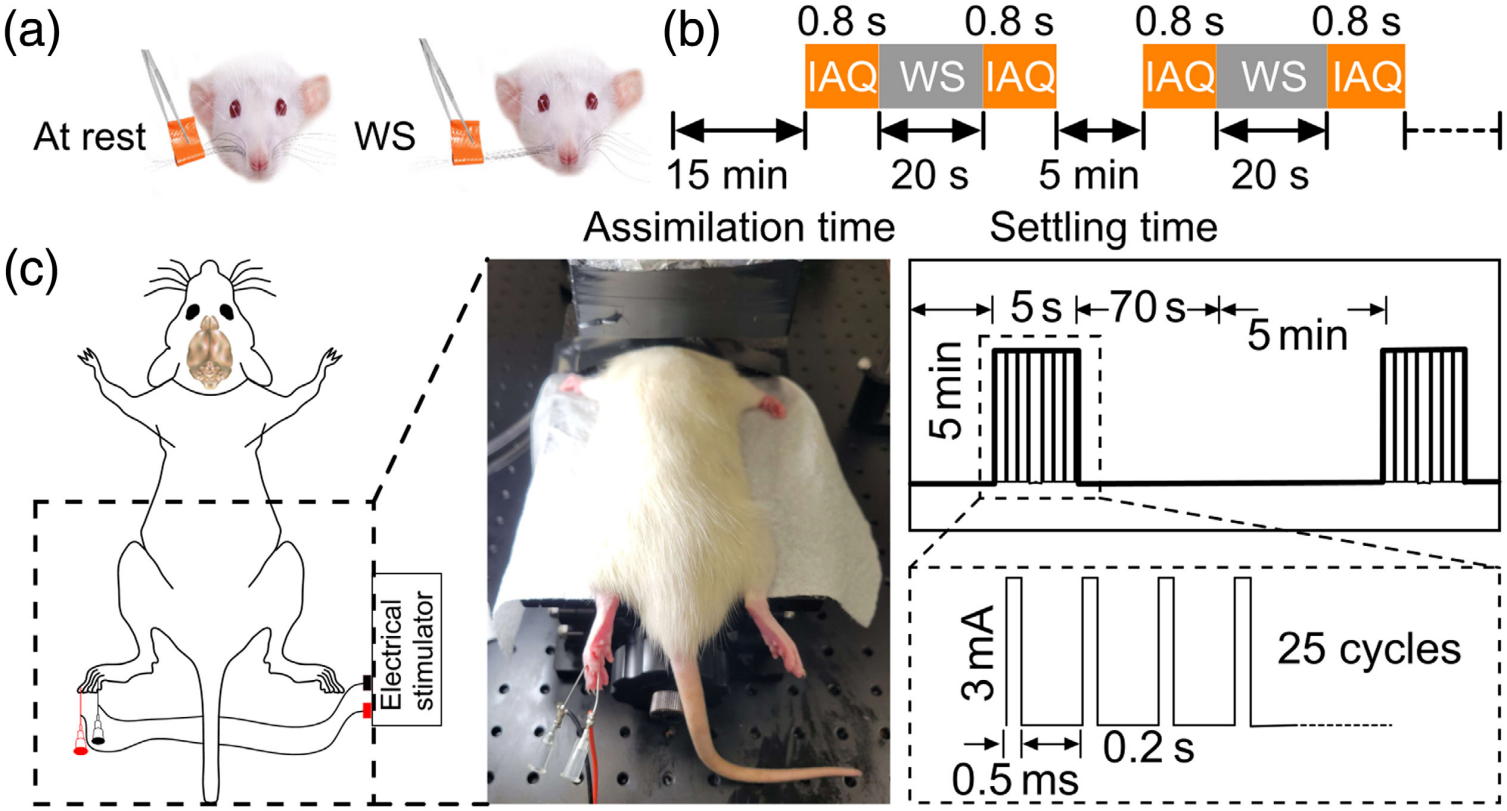
Animal protocols for whisker stimulation and electrical stimulation. (a) Method for stimulating whiskers. (b) Protocol for whisker stimulation and image acquisition. (c) Placement of electrodes on the second and forth index finger of rat’s left foot for electrical stimulation (left) and protocol for electrical stimulation (right). WS, whisker stimulation and IAQ, image acquisition.

### 2.4 Lucy–Richardson Deconvolution Algorithm

Another limitation of any PAM setup is that due to the imperfection of the optical components, reaching the theoretical spatial resolution is not possible. Such distortion in resolution appears as blurriness in the PA image. One potential solution to improve image quality is to estimate the point spread function (PSF) of the system and deconvolve it from the PA images. However, this method only reduces the static aberration and not the dynamic aberration introduced by the sample.^65^ In an imaging system, the distorted output image g(x,y) can be modeled as the convolution of an undistorted input image f(x,y) with a transfer function, or PSF, of the imaging system h(x,y):

g(x,y)=f(x,y)*h(x,y). (6)

The Lucy–Richardson algorithm works based on calculating the maximum-likelihood solution for recovering an undistorted image that has been blurred by a known PSF [see Eq. (7)]:

$$f_{m+1}(x,y)=f_{m}(x,y)[h(-x,-y)*[\frac{g(x,y)}{h(x,y)*f_{m}(x,y)}]],$$ (7)

where $f_{m}(x,y)$ is the estimate of the undistorted image in the m’th iteration. The optimization process starts with $f_{0}(x,y)=g(x,y)$ and iteratively modifies $f_{m}(x,y)$. Following our previous work,^66^ we imaged a very small object (nonfunctionalized colloidal polystyrene microspheres [Alpha Nanotech Inc., United States) of diameter 40  μm] in a gelatin phantom to estimate the PSF of the imaging system. We then utilized the Lucy–Richardson deconvolution algorithm to deconvolve the PSF from the PAM image. For testing the performance of the deconvolution algorithm, a fine branched leaf phantom (Etsy, New York, United States) was imaged.

### 2.5 Preparation of Animal Model

Six-week-old male Sprague Dawley rats (Charles River Laboratories, Wilmington, Massachusetts, United States) were used for this study. Because of the aberrating effect of skull, scalp was removed, and the skull was thinned surgically as follows. Rats were first anesthetized using 4% isoflurane inhalation followed by maintenance doses of 2% to 3% isoflurane during surgery (Doctor Oxygen Service, Inc., Franklin, Wisconsin, United States). Hair was removed using a trimmer to expose the scalp. Next, the scalp was removed from the entire dorsal area. Periosteum was also removed by carefully scraping the exposed skull bone using a scalpel blade. The skin was trimmed laterally, and the temporal muscles were gently detached from the bone on both sides of the skull. The fronto-parietal bone was thinned down ($2.5\;{\mathrm{cm}}^{2}$ over the somatosensory cortex) at low speed with a very fine tip dental drill bit (Dremel 8260, Dremel, New York, New York, United States). The remaining thickness of the bone was ∼0.5  mm from the brain surface. To prevent overheating of the skull, saline was applied repeatedly between the drilling sessions until the skull became flexible, and the major vessels became visible through the wet bone. The animal was introduced to the imaging setup and was kept there for 10 min prior to imaging, to assimilate to the conditions. Ultrasound gel was applied on the exposed region of the skull for acoustic coupling. The experiments were accomplished while the animals were under anesthesia, and the animals were euthanized upon completion of the study. All procedures were conducted in accordance with the “Guide for the Care and Use of Laboratory Animals” and approved by the Wayne State University and University of Illinois Institutional Animal Care and Use Committees.

### 2.6 Animal Protocols for Whisker Stimulation, Electrical Stimulation, and Hemorrhage Induction

We tested our system using whisker and electrical stimulations, some of the most used techniques for studying cerebral hemodynamic activities in animal models.^5^^,^^33^^,^^67^^,^^68^ The whisker stimulation protocol, shown in Fig. 5(a), was as follows: the whiskers from one side of the nose were deflected from the whisker pads by means of a tweezer for 20 s, followed by image acquisition, taking 0.8 s. To obtain the strongest functional signals correlated with whisker stimulation, all whiskers on one side of the nose were taped together and deflected simultaneously from the whisker pad. The rat was then rested for 5 min before repeating the next whisker stimulation, to allow hemodynamic changes to subside and return to baseline levels, thereby not interfering with the subsequent image acquisition. The experiment was repeated for four trials. In the electrical stimulation protocol, a pair of metallic needle electrodes (JM needles, Japan) were inserted to the second and fourth finger of the animal’s left hind paw [see Fig. 5(c)]. A 3 mA pulse with 0.5 mA pulse width and 5 Hz frequency was generated from a function generator (ATF20B, ATTEN Instruments, Columbus, Ohio, United States) to provide electrical stimulation. The stimulation protocol was 5 s on followed by an extended resting period [see Fig. 5(b)]. The image acquisition protocol included continuous imaging from 10 s before stimulation until signal returned to baseline (∼75  s). We also mimicked a brain injury model in rats. For this experiment, the entire skull was removed. Subsequently, we inflicted a small incision in one of the brain vessels using a 1 mm pointed needle (91-01-01, Master Appliance Corp., Racine, Wisconsin, United States).

## 3 Results

We compared the performance of our spiral scanning scheme with unidirectional and bidirectional scanning methods. For the spiral pattern to exhibit the same parameters as the unidirectional and bidirectional scans, the velocity of spiral scanning ($V_{s}$) was kept equal to $V_{\mathrm{tri}}=V_{\text{saw}}$. This consequently achieved Δθ=Δx (separation between scanning points) and, Δr=Δy (line separation or spiral pitch). The length of the square imaging area for the triangular and sawtooth scanning patterns L was adjusted to cover the same total area as the spiral pattern with radius r (eg $L^{2}=\pi {r_{\text{end}}}^{2}$), where $r_{\text{end}}$ is the radius of the circular imaging area produced by the spiral scan shown in Fig. 1(b), and L is the raster scan length.

**Fig. 6:**
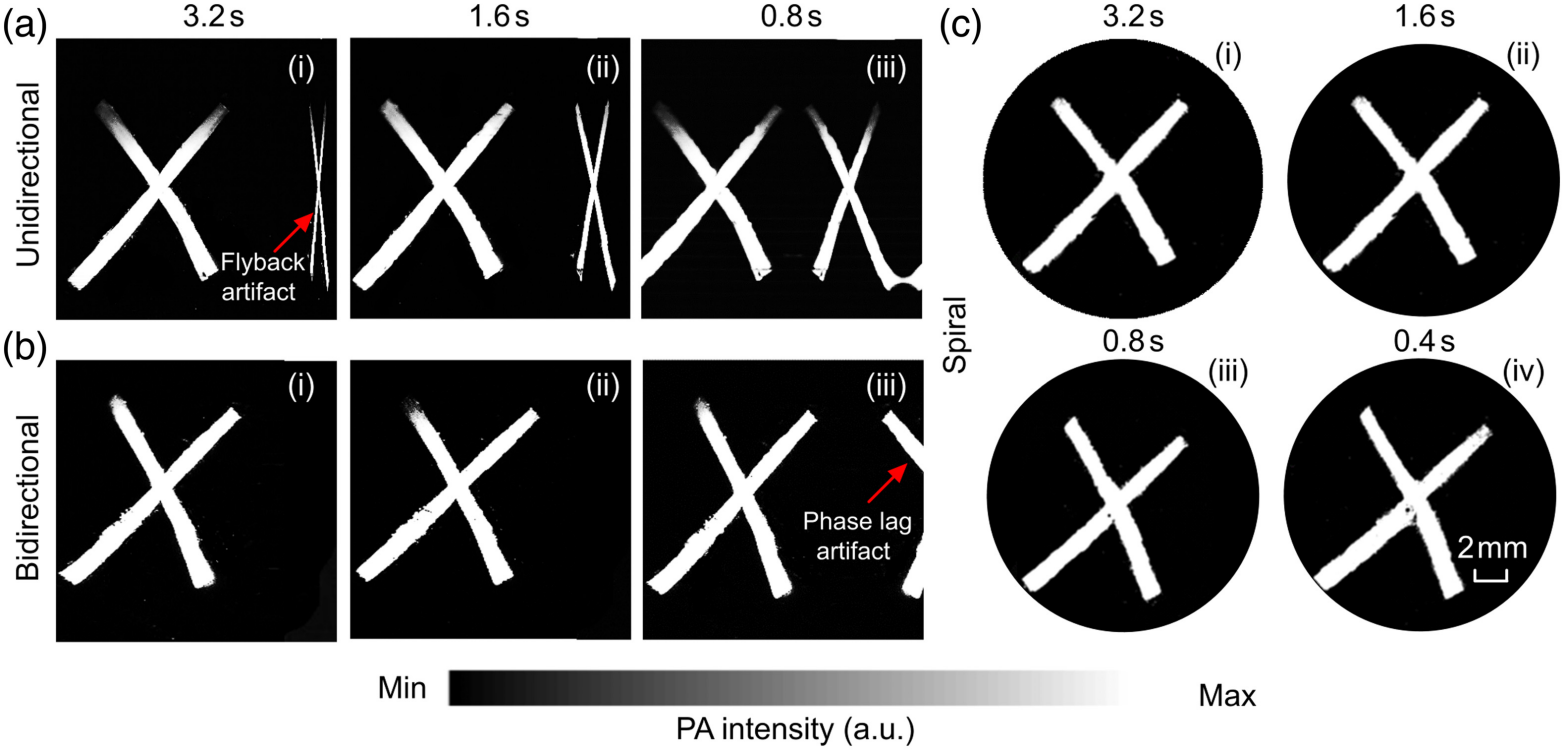
Comparison between the performance of (a) unidirectional, (b) bidirectional, and (c) spiral scans (FOV was square (16  mm×16  mm) for unidirectional and bidirectional scans and circular (diameter 18 mm) for spiral scans in LS-PAM when a “X” shaped phantom made of black tape, was imaged at scanning rates of (i) 3.2  s/frame, (ii) 1.6  s/frame, (iii) 0.8  s/frame, and (iv) 0.4  s/frame.

We imaged a cross, composed of two pieces of black tape, at different frame rates (length: 16 mm). For all three scanning mechanisms, we kept the overall number of scanning points equal (i.e., 160,000 points) to maintain the spiral pitch of 40  μm. With unidirectional scanning, we imaged a frame in 3.2, 1.6, and 0.8 s, as shown in Figs. 6(a)(i)–6(a)(iii). Due to the unidirectional scanning mechanism, a reverse directional image artifact (due to flyback time delay) was introduced, which became more prominent as we scanned at a faster frame rate [see Fig. 6(a)(ii) and 6(a)(iii)]. In contrast, with bidirectional scanning, the problem of reverse directional image artifact was solved [see Fig. 6(b)(i) and 6(b)(ii)]; however, with faster frame rates, the problem of scan misalignment (due to inertial time delays or phase lag delays) became evident, as shown in Fig. 6(b)(iii). With spiral scanning, we were able to scan as fast as 0.4 s [see Fig. 6(c)(iv)] but some distortions including a small blank area were evident.

The distortions that we see at the center of the image are a drawback of the constant velocity spiral scanning method used in this study. Such distortions are hardware-dependent [concerning, for example, the specifications of the galvo mirrors and the data acquisition system (DAQ)]. We refer readers to Ref. 58, which gives mathematical and visual details of distortions associated with spiral scanning. The severity of these distortions is proportional to frame rate of image generation, as seen in Fig. 6(c)(i)–6(iv). For our implementation of constant velocity spiral scanning, we have observed that in the center, the rate of trigger is too fast for the DAQ system, and thus data acquisition does not take place. This malfunction generates zero intensity data, which appears as blank regions in the center of the image [Fig. 6(c)(iii) and 6(c)(iv)]. In addition, at these high frequencies, the galvo mirrors lock themselves and the light beam is not directed toward the target (within the FOV). For instance, if the frame rate was 0.8  s/frame, the initial frequency would have been 20 kHz, and the galvo would be locked until the 189th point’s frequency (that is ∼1  kHz), creating a blank circle of diameter ∼70  μm. If the frame rate was 0.4  s/frame, the initial frequency would have been 40 kHz, and the galvo would be locked until the 388th point’s frequency (that is ∼1  kHz), creating a blank circle of diameter ∼200  μm. Once the DAQ is triggered, if the frequency is still too high for the galvo, the galvo cannot follow accurately, and a distortion similar to the one shown in Fig. 5 of Ref. 58 will also appear near the center of the image. The diameter of the blank region artifact and the nearby distortion decreases with decreasing frame rate. This artifact in many applications, if it is small, can be ignored.

**Fig. 7:**
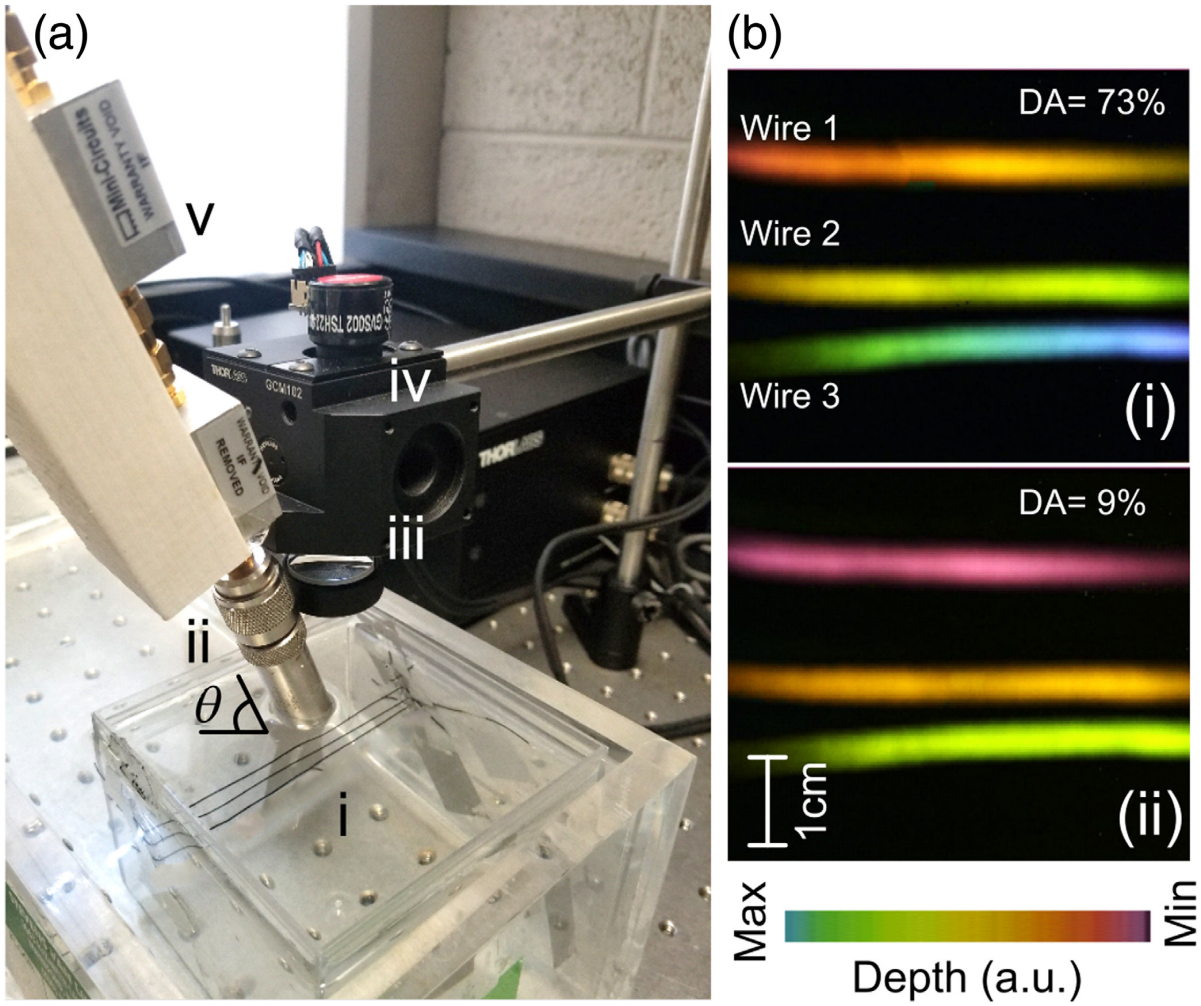
Depth correction algorithm results. (a) Experimental setup to evaluate the depth correction algorithm by imaging a three-wire phantom in water with an ultrasound transducer at an inclined angle of 30 deg. The setup includes: (i) water tank, (ii) ultrasound transducer, (iii) objective lens, (iv) x−y galvo scanner, (v) two-stage low noise amplifier. (b) Photoacoustic images: (i) PAM image of the phantom without depth correction and (ii) PAM image of the phantom with depth correction. DA, depth anomaly.

To evaluate the depth correction algorithm, we prepared a test phantom consisting of three copper wires (diameter 0.5 mm) at different depths as shown in Fig. 7(a). Next, Fig. 7(b) shows the photoacoustic image of this phantom before [Fig. 7(b)(i)] and after [Fig. 7(b)(ii)] depth correction. The averaged depth anomaly, the slope of line profile in the axial direction, was improved from 73% to 9%. The depth anomaly was not improved to zero due to manual error in transducer angle measurements. This algorithm was incorporated into all future results.

**Fig. 8:**
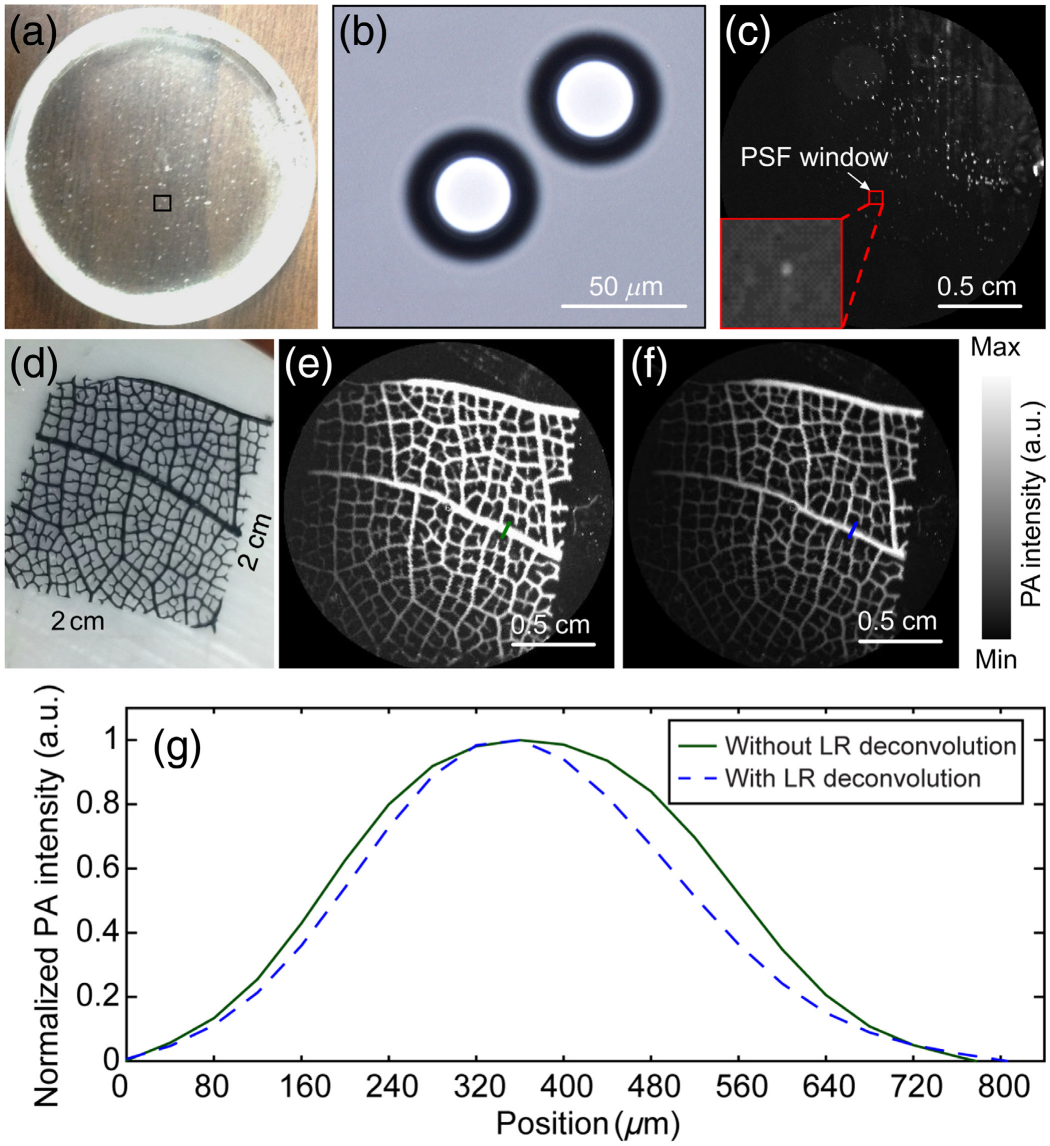
Deblurring algorithm results. (a) A photograph of polystyrene microspheres embedded in gelatin phantom, (b) brightfield photograph of polystyrene microspheres, (c) PAM image of microspheres in gelatin phantom, (d) photograph of a leaf phantom, (e) PAM image before deconvolution, (f) PAM image after deconvolution, and (g) averaged intensity line profiles obtained from the images in (d) (green) and (e) (blue). LR, Lucy–Richardson.

To obtain the system PSF, we imaged nonfunctionalized colloidal polystyrene microspheres (Alpha Nanotech Inc., Vancouver, Washington, United States), of diameter 40  μm, embedded in 8% gelatin in a water phantom as shown in Fig. 8(a). A brightfield image of two microspheres is shown in Fig. 8(b) (we used a 40X-2500X Kohler compound microscope) and the PAM image of microspheres in the gelatin phantom is shown in Fig. 8(c). From the PAM image, we selected a window of 20×20  pixels, enclosing the image of a single microsphere [shown in red box in Fig. 8(c)], representing the PSF of the system. We imaged a leaf phantom as shown in Fig. 8(d) and applied the Lucy–Richardson deconvolution algorithm to correct for image aberrations. The images before and after deconvolution are given in Figs. 8(e) and 8(f), respectively. Line profiles across the line indicated in Figs. 8(e) (in green) and 8(f) (in blue) were compared. The results showed a considerable reduction in the image blurriness after applying the deconvolution algorithm. Therefore, this deconvolution algorithm was incorporated into all future results.

**Fig. 9:**
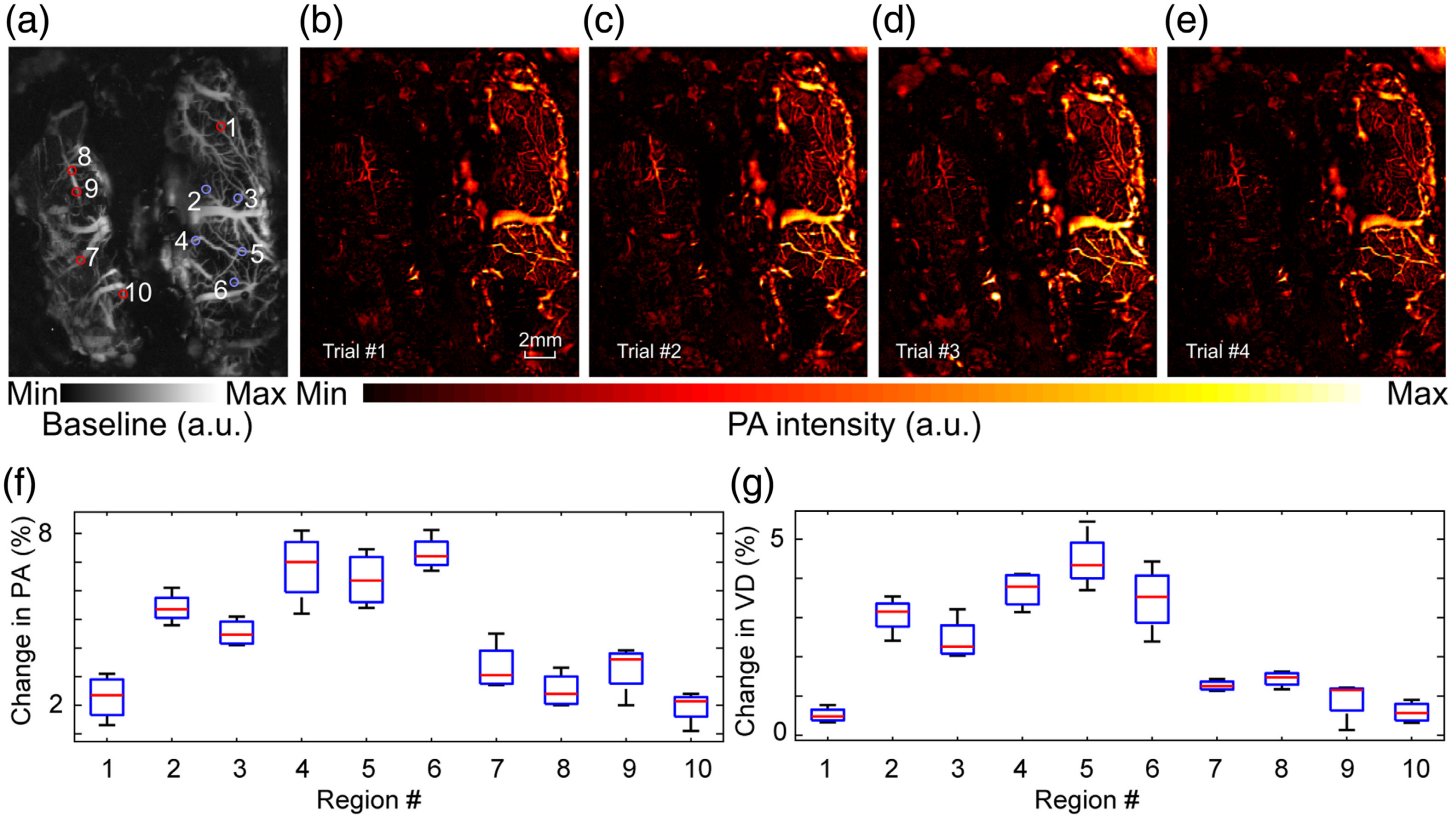
Evaluation of the functional brain imaging capability of sLS-PAM with whisker stimulation. (a) Image of rat’s brain before whisker stimulation. (b)–(e) Activation maps of four separate trials showing an increase in pixel intensity mainly on the right side of the rat brain (left whiskers were stimulated). Maps were developed by subtracting each trial’s PA image after stimulation from each trial’s PA image at rest, graphs showing (f) change in pixel intensity and (g) vessel diameter before and after whisker stimulation for ten separate regions of interest identified on the images in (a).

To evaluate the functional brain imaging capability of our sLS-PAM, we imaged a rat brain *in vivo* during intermittent rest and stimulation periods. All of the following experiments were performed by spiral scans with an imaging time of 0.8 s per frame for an 18 mm diameter area. Whiskers and electrical stimulation were performed. The cycle of stimulation and rest periods was repeated 4 times (4 trials). Using the PAM images of the rat’s brain vasculature at rest [See Fig. 9(a)] and whisker stimulation, stimulation maps were generated by subtracting each trial’s PA image after stimulation from each trial’s PA image at rest. The four stimulation maps are shown in Figs. 9(b)–9(e). By stimulating the rat’s whiskers, we anticipated a functional change in the somatosensory cortex^69^ (because of the light illumination used in PAI, the visual cortex may have been stimulated as well). Because left nose whiskers were stimulated and nerves cross upon entering the brain (decussation), we expected to see functional changes in the right side of the rat’s brain. Although there are other areas showing increase (or decrease) in activity, such changes might be due to functional connectivity between different subsystems. In summary, the changes in the averaged PA signal in the activated brain regions are consistent with the stimulation activity described in the protocol. For quantitative purposes, we selected 10 regions of interest containing vasculature and performed pixel intensity and vessel diameter analyses before and after whisker stimulation. These results are shown in Figs. 9(f) and 9(g), respectively.

**Fig. 10:**
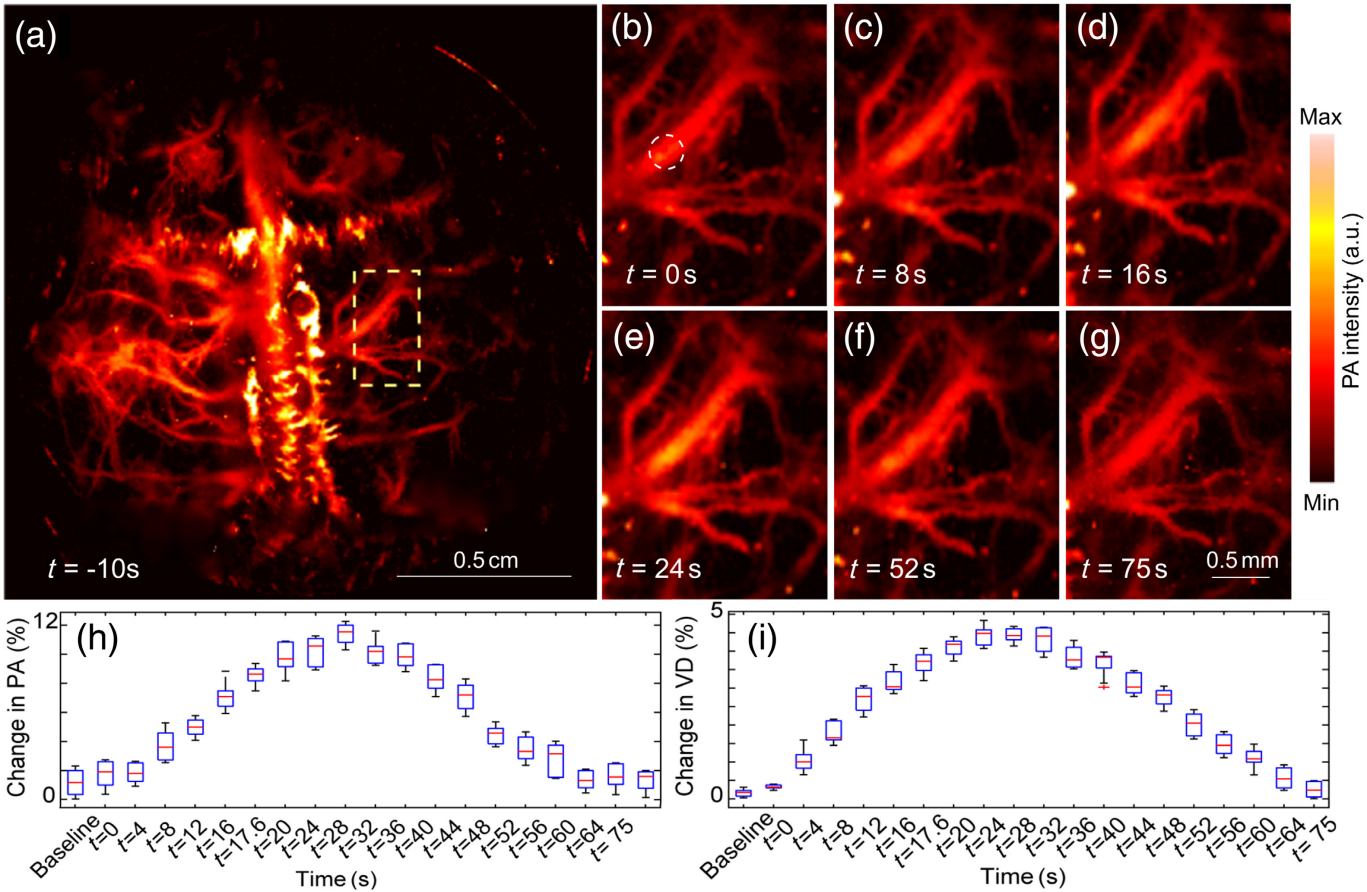
sLS PAM images of rat brain with electrical stimulation: (a) sLS-PAM image at t=0 (beginning of electrical stimulation); (b)–(g) enlarged area of sLS-PAM image denoted by yellow dotted inset, at (b) t=0  s (resting state), (c) t=8  s, (d) t=16  s, (e) t=26  s, (f) t=52  s, and (f) t=75  s; (h) percent change in average pixel intensity over area shown in (b)–(g), over time; and (i) percent change in vascular diameter over time [vessel identified by dashed circle in (b)].

Electrical stimulation was also performed, and images were acquired continuously (see Fig. 5). A PAM image of the whole rat brain is shown in Fig. 10(a). Figures 10(b)–10(g) shows an enlarged version of the inset box in Fig. 10(a), at a range of times. We note increased pixel intensity (over baseline) at the onset of electrical stimulation at t=0 Fig. 10(d). At t=16, we observed some vessels in the enlarged regions become activated, denoted by a further increase in pixel intensities, and the effect of stimulation became more pronounced at t=26  s. The effect of electrical stimulation persisted for about 26 s before starting to diminish. Finally, at 75 s, we see the same intensity as prior to stimulation [Fig. 10(g)]. Figure 10(h) shows the change in the hemodynamics in terms of PA signal intensity from 10 s before to 75 s after stimulation, and Fig. 10(i) shows change in vessel diameter for a single vessel [shown in Fig. 10(b)]. The experiment was repeated 10 times and the rat was rested for 5 min between experiments to allow hemodynamic changes to subside and return to baseline levels. The extent of electrical stimulation was fairly strong (500  μs pulses of amplitude 3 mA delivered in 5 s) compared with a recently reported rodent brain PA imaging system^70^ (250  μs of amplitude 2 mA delivered over 20 s). The rationale for using the more intense stimulation for this study was to demonstrate the capability of the system to capture a prolonged animal response with high time resolution, which we were not able to accomplish with a simpler whisker stimulation protocol, and gentler electrical stimulation.

**Fig. 11:**
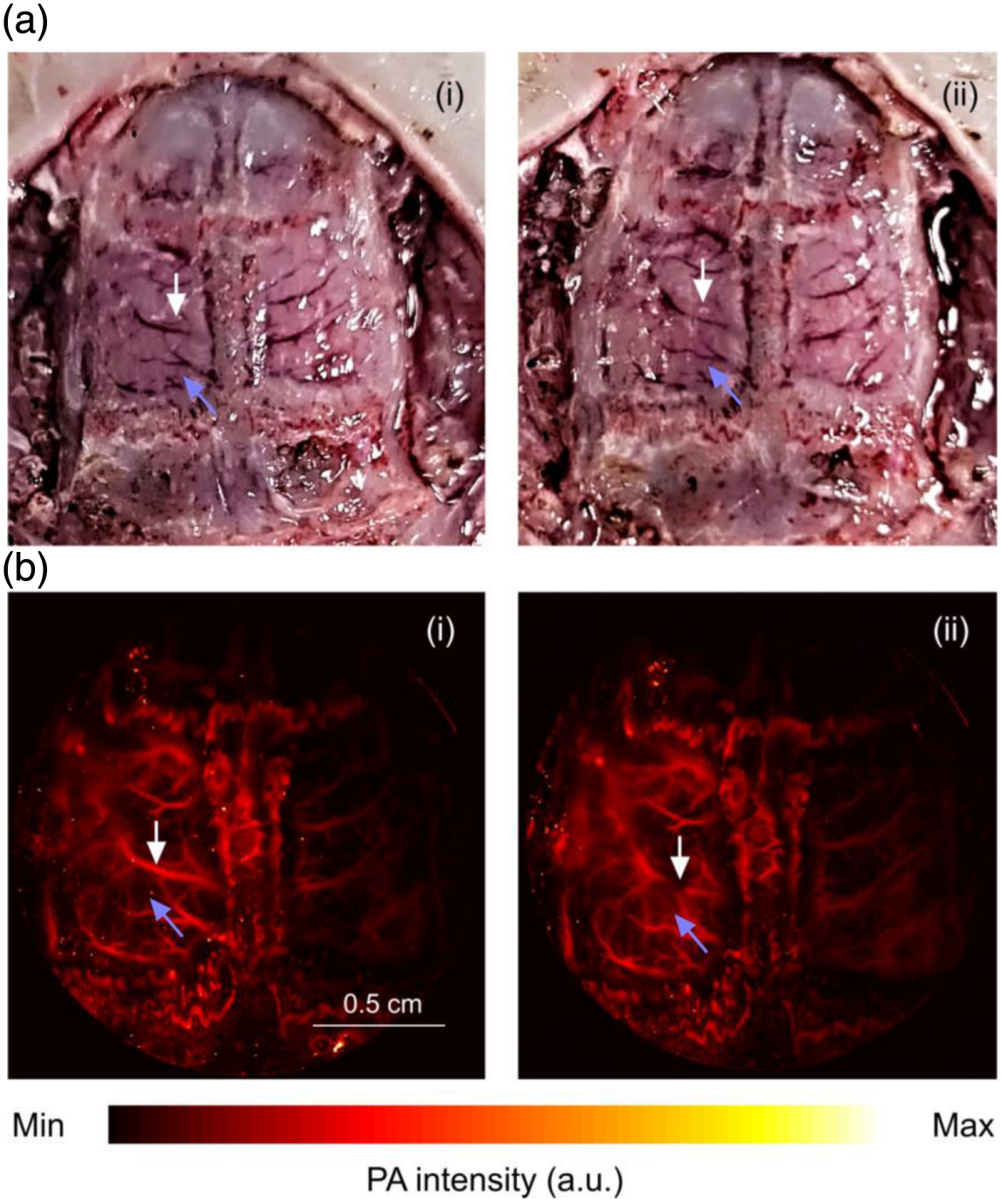
Brain injury model. (a) (i) Rat brain prior to vascular incision and (ii) rat brain after vascular incision. (b) (i) sLS-PAM image before vascular incision and (ii) sLS-PAM image after vascular incision. White pointer detonates injured area and purple pointer denotes area with blood leakage.

We also mimicked a brain injury model in a rat by vascular incision. For this experiment only, the rat skull was entirely removed. The rat brain before and after vascular incision is shown in Figs. 11(a)(i) and 11(a)(ii), respectively. The sLS-PAM images before [Fig. 11(b)(i)] and after vascular injury [Fig. 11(b)(ii)] were acquired and the injured area was clearly distinguishable, as marked by a white pointer. Furthermore, blood leakage around the injured area was observed, specified by a purple pointer.

## 4 Discussion

Neuroimaging techniques play a crucial role in exploring functional brain activity by measuring cerebral hemodynamic parameters and enhancing our fundamental understanding of neuroscience. Rats have become extensively utilized in neuroscience research due to the availability of numerous transgenic rat models tailored for various studies. In our previous research, we investigated brain activities by employing ring array PACT to image the primary functional cortical regions. As a result, we were inspired to conduct the current study with the goal of extending this imaging capability to visualize finer regions, i.e., the cortical subregions within the main functional regions. We developed a system which we call sLS-PAM, with a sufficiently high resolution, fast frame rate (>1  Hz), and wide FOV (∼18  mm) for visualizing the vasculature and hemodynamics in an entire rat brain.^71^ Our system can work continuously we have tested is use imaging phantoms for up to 5 min and the optics were intact and showed no damage. Moreover, it appears reasonably safe for animals, as we saw no sign of damage to the rat’s brain, even after 75 s of continuous imaging (and 10 consecutive imaging session each lasting over 75 s). Although we used a 2.25 MHz Centrascan transducer, a higher frequency transducer with the same or higher sensitivity would improve the lateral resolution of the current system, and we are working with a company to develop a customized transducer for this purpose. Although we do not claim our system is the fastest reported in the literature, it is fast enough for rat brain functional imaging applications. To demonstrate its place among other designs, we list in Table 2 some existing OR-PAM systems with their imaging area, temporal resolution, and temporal resolution per imaging area, compared to our system (written in bold).

**Table 2:** Comparison between OR-PAM systems. Characteristics of our system are written in bold.

Ref.	Method	Imaging area (${\mathrm{mm}}^{2}$)	Temporal resolution (s)	Temporal resolution per imaging area ($s\;{\mathrm{mm}}^{-2}$)
47	Water immersible MEMS scanning PAM	2 × 5	1.25	0.13
49	Fully motorized scanning PAM	8 × 10	180	2.25
50	Waterproof 2D galvanometer scanning PAM	8 ×13	100	0.96
51	Cylindrical focused wide field PAM	4.5 × 1	1	4.5
52	Wide field polygon scanning PAM	12 × 12	5	0.03
72	Semi-water immersible galvanometer scanning PAM	12.9 × 8 mm	15	0.15
73	LS-PAM with ZnO detector	2 × 2	1.7	0.43
74	Fiber optics-based PAM	8 × 13	32	0.31
75	LS-PAM with transparent detector	20 × 20	180	0.45
61	Water immersible 12 facet polygon scanning PAM	11 × 7.5	0.5 (unprocessed)	0.006
This report	**sLS-PAM**	**Diam = 18 cm (circular)**	**0.8**	**0.003**

Some of the limitations of sLS-PAM are as follows. Because of the inclined angle of the transducer, the depth-resolved information needs correction. Another shortcoming is that the angle and distance between the transducer and the imaging target needs optimization for each application to ensure adequate sensitivity and FOV. These concerns may be resolved by motorizing the transducer holder. Finally, at very fast imaging speeds, the current implementation of the spiral scanning system has distortion and a blank area artifact in the center of the image, due to high angular frequency at scan inception. This can be overcome by developing a distortion correction algorithm.

## 5 Conclusion

We developed a high-resolution, fast frame rate, and wide FOV OR-PAM spiral scanning system. We do not claim the system has the fastest frame rate, highest resolution, largest FOV, and highest sensitivity altogether, but we demonstrated it is capable of imaging a circular region with 18 mm diameter in 0.4 s with negligible artifact (i.e., a small blank circle in the center of the image) and some distortion, or in 0.8 s virtually artifact free with negligible distortion, which is ample for the purpose of imaging vasculature and hemodynamics in a rat brain. We demonstrated the functional imaging capability of the sLS-PAM system by imaging cerebral hemodynamics in response to whisker and electrical stimulation and used it for vascular imaging of a modeled brain injury. We believe that there are many neuroscience questions that can be answered by the described system.

## Data Availability

Code and Data can be made available upon reasonable request.
